# Looking forward in 2021

**DOI:** 10.1038/s42003-021-01718-w

**Published:** 2021-02-19

## Abstract

A new year symbolizes new hope for the future, especially this year as we start to see the first wave of vaccines administered against COVID-19. Here, we take stock of the year behind us and look forward to seeing where science takes us in 2021.

2020: It was the best of times, it was the worst of times. Of course, the “worst” comes to mind much more easily. In barely more than a year, we’ve seen a novel coronavirus become a pandemic in the blink of an eye. Wildfires ravaged Australia and much of the Western United States. There were more named hurricanes than letters of the alphabet. Heatwaves shattered records in many places, including parts of India that reached 50 degrees Celsius. Not to mention the economic and political crises that defined the year. Put another way, 2020 felt like a rather long decade.

The past year also brought much-needed hope. Vast numbers of scientists across the globe rose to the challenge of COVID-19, producing an impressive amount of knowledge and, amazingly, developing and delivering safe, effective vaccines. Labs across the biological sciences contributed to the pandemic management in countless ways, 3D printing ventilators or contributing spaces and equipment for COVID-19 testing. And all the while, despite lab closures, illnesses, caretaking responsibilities, supply shortages, and the general emotional exhaustion wrought by 2020, scientists—regardless of sub-discipline—continued to advance science.

Like everyone else, we are very happy to have 2020 firmly behind us. But we also want to acknowledge some of the positive aspects of the last year before looking ahead to what 2021 may have in store.

In the early months of 2020 (and in some places even earlier) as lockdowns went into place, many researchers found themselves with more time to write. This translated into a surge of manuscript submissions to journals across the spectrum, including *Communications Biology*. While a good chunk of these papers were focused on learning as much as possible about the novel coronavirus, the majority were unrelated to the pandemic. We are humbled and grateful to see the research community continue to support each other by editing and reviewing manuscripts while enduring their own hardships due to the pandemic. In an accompanying editorial^1^, we thank each of the 5314 individuals who reviewed articles for us last year.

We are humbled and grateful to see the research community continue to support each other by editing and reviewing manuscripts while enduring their own hardships due to the pandemic.

We are also grateful to have published so many exciting papers in 2020. On the COVID-19 front, we published a study that compared ACE2 variants between humans and non-human primates and found that many of our closest relatives are also at risk of contracting the disease^2^. In a review article, Chakravarty et al. explored the greater severity of COVID-19 in men, and how the virus may affect the treatment of prostate cancer in men in the highest risk group^3^.

Our most-downloaded and most talked-about paper of the year was from Cameron Radford et al.^4^, who showed that painting artificial eyespots on the backsides of cows can deter large predators. Our most-cited paper of 2020 so far reports a computational toolkit, BrainSpace, for analyzing functional MRI datasets^5^. Some of the papers we published also made the news, including the discovery of a new type of stinging-cell structure in a jellyfish^6^ (covered in the New York Times here) and the surprising finding that microbes are thriving in 30–100 million-year-old basaltic rock beneath the seafloor^7^ (covered in the Atlantic here). Of course, this is just a taste of the 735 research articles published in *Communications Biology* last year.

Looking forward to the rest of 2021 and into the future, we’re confident that the pursuit of knowledge will continue to amaze us. We anticipate, and hope, for breakthroughs that will help us to solve our other shared dilemmas: climate change, hunger, and the continued destruction of the world’s wild places. As a journal, we remain committed to our mission of serving the needs of the life sciences research community by ensuring a fair and rigorous review process. We also see it as our duty to create an inclusive environment for all biologists, which is why we have pledged to raise the proportion of women among our reviewers, to maintain a high level of diversity in our editorial board, and to raise the profile of scientists who belong to underrepresented groups by various means, including the publication of interviews with exceptional scientists. We have started off the fourth year of *Communications Biology* with a Q&A article featuring an interview with Dr. Lorin Crawford^8^ about his research at the intersection of machine learning and biology. We plan to publish many more Q&As this year with the goal of highlighting the great diversity of researchers in our community.

As we’ve all learned, the future is impossible to predict. But we’re hopeful that 2021 will be a better year for us all.
